# Intracranial Acute Subdural Hematoma Following Spinal Anesthesia for Cesarean Delivery

**DOI:** 10.1002/ccr3.70992

**Published:** 2025-09-24

**Authors:** Abraham Fessehaye Sium, Tizita Abraham Basha, Fenta Mollaligne, Delayehu Bekele

**Affiliations:** ^1^ Department of Obstetrics and Gynecology St. Paul's Hospital Millennium Medical College (SPHMMC) Addis Ababa Ethiopia

**Keywords:** postspinal headache, postspinal subdural hematoma, spinal anesthesia, subdural hematoma

## Abstract

Intracranial subdural haematoma is a rare but serious complication of spinal anesthesia in obstetric patients undergoing Cesarean Delivery (CD). In this case series, we analyzed 3 patients who developed acute intracranial subdural hematoma following spinal anesthesia for CD. Clinical presentation with headache of 3–4 weeks duration that worsened over 2–3 days prior to admission for neurosurgical intervention was evident in all the patients. Brain CT‐Scan was the imaging modality used to confirm the diagnosis of acute intracranial subdural hematoma in all of them, and Burr hole was done to evacuate the hematoma, and all of them fully recovered with no neurological deficit. Their postoperative follow‐up visit after 2 weeks didn't document any neurologic abnormality. Postspinal headache that persists beyond 2 weeks during the postpartum period warrants an urgent neurosurgical evaluation, as it may be due to subdural hematoma.

**Summary**

- This case series highlights the importance of having a high index of suspicion for intracranial subdural hematoma in patients that present with postspinal headache or severe global headache.
- Complete neurological evaluation and work‐up for patients with no predisposing risk factor that present with postspinal headache persisting beyond 2 weeks and non‐postural global type of headache (regardless of its duration) should be performed, as it may be due to subdural hematoma.

## 1 Introduction

Post‐dural puncture headache (PDPH) is a common side effect of Spinal Anesthesia and is defined as a postural headache that appears or intensifies after 15 min of standing which improves on lying down, according to the International Headache Society's diagnostic criterion for PDPH [1]. Two mechanisms cause this headache: shifts of intracranial contents and gravitational traction on pain‐sensitive structures due loss of cerebrospinal fluid (CSF) through the dural puncture [2] and compensatory vasodilation in response to the low intracranial pressure [3]. Additional symptoms that can occur in more than 50% of patients include nausea, tinnitus, vertigo and photophobia [4]. Symptoms often resolve spontaneously within 5 days or when treated with analgesics and bed rest. More than 85% of PDPH resolve within 6 weeks [5].

Intracranial subdural haematoma is a rare but serious complication of dural puncture following epidural and spinal anesthesia in the obstetric population [6, 7], that mimics PDPH in terms of clinical presentation [8]. Caudal displacement of the brain due to large CSF spillage leading to stretching of the fragile bridging veins that connect the cortex with the venous sinuses, which ultimately rupture and bleed, is the postulated pathogenesis of this rare spinal anesthesia complication [9]. In general, there is a scarcity of evidence on intracranial subdural hematoma after spinal anesthesia for cesarean delivery. The most recent and biggest review of the literature on this topic to date identified 20 case reports [10]. A recent case series also described the clinical course and treatment outcomes of this devastating complication of spinal anesthesia in 6 obstetric patients [11]. We present a second case series on this topic, which sought to describe the clinical presentation and neurologic outcomes of postspinal acute intracranial subdural hematoma following neurosurgical treatment.

## 2 Case History/Examination

This case series analyzed women with acute intracranial subdural hematoma following spinal anesthesia for cesarean delivery over 10 years (2013–2023) who were managed with a neurosurgical intervention at St. Paul's Hospital Millennium Medical College (SPHMMC), Ethiopia, Addis Ababa. Data were collected through chart review. A formal ethical clearance letter was obtained from the Institutional Board Review (IRB) of St. Paul's Hospital Millennium Medical College (Addis Ababa, Ethiopia). The requirements for written informed consent were waived by this IRB.

Table 1 presents the clinical characteristics, extent of neurosurgical intervention, and procedure outcomes of cases of acute subdural hematoma following Cesarean Delivery (CD). There were 3 cases of acute subdural hematoma following CD during the study period. The patients' age ranged from 27 to 30 years. All cases presented with persistent postspinal headache of 3–4 weeks duration, following cesarean delivery, that worsened over the last 2–3 days before presentation, accompanied by vomiting. Repeated attempts of dural puncture for administration of spinal anesthesia were evident in all cases. Lidocaine was administered in one of the cases while bupivacaine was the drug used as spinal anesthesia in the other 2 patients. All patients presented without either coagulation disorders or anticoagulant/antiplatelet therapy. There was no history of trauma in any of the cases.

**TABLE 1:** Clinical presentation, neurosurgical intervention, and procedure outcomes, Ethiopia (2013–2023).

Variable	Case 1	Case 2	Case 3
Maternal age	27	30	30
Presentation	Severe global headache with worsening over 3 days with accompanied with vomiting	Severe global headache with worsening over 3 days with accompanied with vomiting	Severe global headache with worsening over 2 days with accompanied with vomiting
Interval between delivery and diagnosis of acute subdural hematoma	22 days	23 days	33 days
History of hypertension or bleeding diathesis	None	None	None
Scenario of spinal anesthesia administration	Lidocaine administered after repeated attempts of dural puncture	– Repeated attempts of administration of bupivacaine into the dural space – General anesthesia was given after failed spinal anesthesia was declared	Successful bupivacaine administration after repeated attempts of administration
Brain‐CT diagnosis	Acute subdural hematoma	Acute subdural hematoma	Acute subdural hematoma
Neurosurgical procedure	Hematoma evacuation through burr hole	Hematoma evacuation through burr hole	Hematoma evacuation through burr hole
Procedure outcomes	Discharged after 3 days of postoperative period with improvement	Discharged after 3 days of postoperative period with improvement	Discharged after 3 days of postoperative period with improvement

## 3 Differential Diagnosis, Investigations and Treatment

The differential diagnosis was postspinal headache, chronic subdural hematoma, preeclampsia, and acute subdural hematoma. After ruling out the other differentials, finally, acute subdural hematoma was confirmed in three of them with a Brain CT scan (Table 1), and a burr hole was the neurosurgical procedure performed to evacuate the hematoma in the patients. Multidisciplinary team composed of obstetricians, internists, and neurosurgeons was involved in the evaluation and the management of the cases.

## 4 Outcome and Follow‐Up

All patients were discharged from hospital after 2–3 days of stay with improvement, with no sensory or motor neurologic deficit. Their postoperative follow‐up visit after 2 weeks didn't document any abnormal findings.

## 5 Discussion

Persistent postspinal headache of 3–4 weeks duration was the hallmark of clinical presentation in the present series, and the history of repeated attempts of dural puncture during spinal anesthesia administration for cesarean delivery was evident in three of the patients. In all the cases, Brain CT‐scan was used to confirm the diagnosis of acute subdural intracranial hematoma, and Burr hole was performed to evacuate the hematoma with good prognosis and no remnant neurologic deficit.

The development of intracranial subdural hematoma following spinal anesthesia is rare, ranging between 1 in 1 million and 1 in 1.5 million, with a fatal complication [12, 13]. A high index of suspicion for the pattern and characteristics of postspinal headache followed by a meticulous neurologic examination is key for the early diagnosis of subdural hematoma [14]. Among 56 reported cases of intracranial subdural hematoma in the obstetric population described in the largest literature review on this topic, 20 cases were post spinal anesthesia administration for cesarean delivery. Predisposing risk factors were identified in only one patient who received enoxaparin 40 mg 4 h after intrathecal injection, while the rest of the 19 patients had no predisposing risk factors. Multiple dural punctures were noted in four out of 20 patients, and only 60% of the patients presented with postdural pucture headache (PDPH) as the first symptom, while another 6 patients had a severe non‐postural headache. Diagnosis of an intracranial subdural hematoma was confirmed by CT scan in 16 patients; in four patients, MRI was performed. Most (13 patients, 65%) needed urgent surgical intervention, and recovery was achieved in 70%, with five patients (25%) suffering residual neurological defects and one patient dying following irreversible severe cerebral edema. Diagnosis was made in 95% within the first month [10], which is similar to a report of a mean of 22 days of presentation with headache by Amorim et al. in a review of 31 cases [15].

In our case series, all patients presented with PDPH of 3–4 weeks (22–33 days) duration and experienced severe non‐postural headache 2–3 days prior to their presentation for neurosurgical evaluation. All patients had a history of multiple dural punctures; apart from this, no predisposing risk factor for subdural hematoma was identified in any of the cases. In all cases, the diagnosis was made with a Brain‐CT scan. All of them were managed surgically with burr hole and none of them suffered residual neurological defects. Similarly, previous case series on this topic identified 6 patients who underwent surgical evacuation of subdural hematoma that developed after spinal anesthesia was provided for Cesarean delivery. Five patients recovered, and one patient died 2 days after surgery. Contrary to our findings, in the study, 2 patients presented with deterioration of consciousness a few hours after delivery, while the rest 4 presented with persistent headache and lethargy within a week after delivery [11].

Strengths of our case series include being among the first case series on the topic of discussion, as the evidence on this topic is mainly derived from case reports. Small sample size and retrospective nature of our data are the main limitations. Not including CT‐scan images for the patients included in the analysis is the other limitation of this series.

In summary, findings of our case series highlight the importance of having a high index of suspicion for intracranial subdural hematoma in patients that present with postspinal headache or severe global headache, irrespective of its duration. We propose neurological evaluation and work‐up for patients with no predisposing risk factors that present with postspinal headache persisting beyond 2 weeks and non‐postural global types of headache (regardless of its duration), as it may be due to subdural hematoma.

## Author Contributions

**Abraham Fessehaye Sium:** conceptualization, data curation, formal analysis, writing – original draft, writing – review and editing. **Tizita Abraham Basha:** conceptualization, data curation, writing – original draft. **Fenta Mollaligne:** conceptualization, data curation, formal analysis, writing – original draft. **Delayehu Bekele:** conceptualization, data curation, formal analysis, writing – original draft.

## Ethics Statement

Formal ethical clearance letter was obtained from the Institutional Review Board (IRB) of St. Paul Hospital Millennium Medical College. The requirement for written informed consent was waived by the IRB.

## Conflicts of Interest

The authors declare no conflicts of interest.

## Data Availability

Data are available from authors upon reasonable request.
